# The Relevance of Nrf2 Pathway and Autophagy in Pancreatic Cancer Cells upon Stimulation of Reactive Oxygen Species

**DOI:** 10.1155/2016/3897250

**Published:** 2015-11-22

**Authors:** Lun Zhang, Jiahui Li, Jiguang Ma, Xin Chen, Ke Chen, Zhengdong Jiang, Liang Zong, Shuo Yu, Xuqi Li, Qinhong Xu, Jianjun Lei, Wanxing Duan, Wei Li, Tao Shan, Qingyong Ma, Xin Shen

**Affiliations:** ^1^Department of Hepatobiliary Surgery, First Affiliated Hospital of Xi'an Jiaotong University, Xi'an 710061, China; ^2^Department of Oncology, First Affiliated Hospital of Xi'an Jiaotong University, Xi'an 710061, China; ^3^Department of General Surgery, Second Affiliated Hospital of Xi'an Jiaotong University, Xi'an 710004, China; ^4^Department of General Surgery, First Affiliated Hospital of Xi'an Jiaotong University, Xi'an 710061, China; ^5^Department of Anesthesiology, First Affiliated Hospital of Xi'an Jiaotong University, Xi'an 710061, China

## Abstract

Nrf2 (NF-E2-related factor 2) pathway and autophagy both can respond to oxidative stress to promote cancer cells to survive in the tumor microenvironment. We, therefore, explored the relevance between Nrf2 pathway and autophagy in pancreatic cancer cells upon stimulation of reactive oxygen species (ROS). Pancreatic cancer cells were cultured under controlled ROS stressing condition or basal condition. Different inhibitors were used to prevent autophagy at particular stages. Nrf2 siRNA was used to inhibit Nrf2 pathway activation. Ad-mRFP-GFP-LC3 infection was used to monitor autophagic flux. The result shows that a small amount of exogenous hydrogen peroxide (H_2_O_2_) can significantly improve the level of intracellular ROS. Moreover, our findings indicate that ROS promotes the activation of both Nrf2 pathway and autophagy in pancreatic cancer cells. Moreover, our data demonstrate that suppression of autophagic activity at particular stages results in an increased promotion of Nrf2 pathway activation upon ROS stimulation. Furthermore, we found that silencing of Nrf2 promotes autophagy upon ROS stimulation. In addition, Nrf2 interference effectively promotes autophagic flux upon ROS stimulation. In summary, our findings suggest that Nrf2 pathway and autophagy have a negative interaction with each other upon ROS stimulation.

## 1. Introduction

Autophagy is an evolutionary conserved lysosomal degradation process in which the cells degrade long-lived proteins, misfolded proteins, and damaged cytoplasmic organelles for recycling [1]. Autophagy has been considered to maintain the cellular homeostasis and adaption to stressed conditions such as oxidative stress, nutrient starvation, and hypoxia [1–4]. For its many important roles, it is not surprising that impaired autophagic function promotes the progression of cancer. However, cancer cells break down cellular damaged organelles and accumulated proteins by autophagy, allowing the catabolites to be recycled and thus used for biosynthesis and energy metabolism to cope with the stressing conditions, which is essential to enable cancer cells to survive [5, 6].

It has been considered that Nrf2, a transcriptional factor, is an adaptive cellular response to protect cells against oxidative stress. Nrf2 is targeted by Keap1 (Kelch-like ECH-associated protein1) for ubiquitylation and proteasomal degradation under normal condition [7]. When faced with cellular stressing signals (e.g., oxidative stress), the interaction between Nrf2 and Keap1 is disrupted, resulting in Nrf2 stabilization and translocation from cytoplasm to the nucleus, which is regarded as canonical way of Nrf2 activation [8]. As a result, the nuclear Nrf2 binds to antioxidant response elements (AREs) or electrophile response elements (EpREs) to deal with the stressing signals [9–11]. High levels of Nrf2 have been observed in many cancers, including head and neck, gall bladder, lung cancer, and colorectal cancer [12–14], which promote the growth and survival of cancer cells under stressing conditions. Nrf2 pathway and autophagy both have the ability to antagonize cellular stressing signals by promoting a series of antioxidant programs. Furthermore, studies have shown that Nrf2 and autophagy both contribute to the chemoresistance [15–18].

The relationship between the Nrf2 pathway and autophagy has been explored in recent years, and researchers found that the association between them mainly relied on p62/SQSTM1, an adaptor for selective autophagy, and Keap1 [19–21]. Inhibition of autophagy leads to accumulation of p62. P62 works to sequester Keap1 into the autophagosomes, inhibiting the ubiquitylation of Nrf2, resulting in the noncanonical activation of Nrf2 [19–21]. Many studies have shown that cancer cells accumulate more reactive oxygen species (ROS) than normal cells [22, 23]. Consistent with other researchers, we found that the level of ROS in pancreatic cancer cells had elevated along with the increasing metastatic ability [24]. With the increased ROS, cancer cells induce antioxidant programs to set a new redox balance, resulting in cellular adaptation. Studies have shown that autophagic inhibitor (chloroquine) caused accumulated ROS in cells, and downstream of Atg1, FIP200-(Atg17 homologue) knockout livers, and Atg5- and Atg7-knockout cells both increase ROS production [25–30]. On the basis of these results, we hypothesized that there is a possibility that autophagy inhibition not only leads to accumulation of p62 to activate Nrf2 pathway by a noncanonical way but also increase ROS production to directly activate Nrf2 pathway.

In addition, another study indicated that Nrf2 could lower the level of intracellular ROS [31]. Thus, we speculated that the relationship between Nrf2 pathway and autophagy could not be a simple upstream or downstream. The mechanism of the interaction between Nrf2 pathway and autophagy is needed to be investigated to facilitate the discovery of new therapies. In this study, we set pancreatic cancer cells at an increased ROS level to simulate oxidative stress condition and explore the relevance between Nrf2 pathway and autophagy.

## 2. Materials and Methods

### 2.1. Cell Culture and Reagents

Human pancreatic cancer cell lines BxPc-3, PANC-1, SW1990, AsPC-1, and MiaPaCa-2 were obtained from and validated by the Cell Bank of the Chinese Academy of Sciences (Shanghai, China) and cultured as per their instructions. Briefly, cells were cultured with a humidified atmosphere of 95% air and 5% CO_2_ at 37°C in Dulbecco's modified Eagle's medium (DMEM) (HyClone, Logan, USA) supplemented with 10% heat-inactivated fetal bovine serum (FBS) added with 100 *μ*g/mL ampicillin and 100 *μ*g/mL streptomycin. In experiments designed to elevate the level of intracellular ROS, H_2_O_2_ was added in the serum-free media with a final concentration of 100 *μ*mol/L for 24 h. Different autophagic inhibitors were used in this study: 3-MA of 5 mmol/L or CQ of 40 *μ*mol/L for 24 h. Antibodies were obtained from the following resources: anti-*β*-actin antibody (Santa Cruz, USA), anti-Nrf2 antibody (Abcam, USA), anti-Beclin1 antibody (Abcam, USA), anti-LC3 antibody (Sigma, USA), and anti-p62 antibody (Proteintech Group, USA). Autophagic inhibitors were obtained from the following resources: 3-MA (HanBio, China) and CQ (Sigma, USA).

### 2.2. Cell Proliferation Analysis

PANC-1 cells were seeded in 96-well plates at the point of 24 h prior to the serum free incubation at an amount of 5–10 × 10^3^ cells per well. Following the serum starvation of 24 h, cells were maintained in medium with concentrations of H_2_O_2_ ranging from 100 to 400 *μ*mol/L. At the time point of 24 h, 48 h, or 72 h, the medium in each well was removed, and then MTT reagent (3-(4, 5-dimethylthiazol-2-yl)-2, 5-diphenyltetrazolium bromide) was added. After incubation of 4 h at 37°C, DMSO was added to each well with an amount of 150 *μ*L. The microplate reader (BIO-TEC Inc, VA) was used to measure the optical densities (OD) at 490 nm. The proliferation rate was determined as OD (cells plate)/OD (blank plate).

### 2.3. Detection of Intracellular ROS

The level of intracellular ROS was detected using an oxidation-sensitive fluorescent probe (DCFH-DA). After treatment with H_2_O_2_ 100 *μ*mol/L or not, PANC-1 cells were washed twice with PBS. Then cells were incubated with 10 *μ*mol/L DCFH-DA for 20 min at 37°C according to the manufacturer's instructions. DCFH-DA was oxidized to the fluorescent compound 2, 7-dichlorofluorescein (DCF) in the presence of ROS. DCF fluorescence was detected by FACScan flow cytometer (Becton Dickinson). 10 000 events were collected for each sample.

### 2.4. RNA Extraction and Quantitative Real-Time PCR

Total RNA was extracted from pancreatic cancer cells by Trizol reagent (Invitrogen, CA, USA) according to the manufacturer's instructions. Reverse transcription was carried out using a PrimeScript RT reagent Kit (TaKaRa, Dalian, China), and the real-time PCR assay was performed with an iQ5 Multicolor real-time PCR Detection System (Bio-Rad, Hercules, CA, USA) and a SYBR Green PCR Kit (TaKaRa) followed by their manufacturer's instructions. The PCR primer sequences of Nrf2, Beclin1, LC3 and *β*-actin are shown in Supplementary Table S1 in Supplementary Material available online at http://dx.doi.org/10.1155/2016/3897250. For all real-time PCR analyses, *β*-actin was used as a normalization control to quantify the relative expression of the target gene.

### 2.5. RNA Interference

Six siRNAs for NRF2 (Supplementary Table  S2) were purchased from GenePharm (Shanghai, China). Cells were seeded in six orifice plates with an amount of 2 × 10^5^ per well and transfected with a final concentration of 100 nM siRNA using Lipofectamine RNAi MAX Reagent (Invitrogen, CA, USA) according to the manufacturer's instructions. After transfection, cells were used for further study at the time point of 48 h.

### 2.6. Western Blot

The protein levels of Nrf2, Beclin1, LC3, and p62 were quantified by semiquantitative densitometric analysis. Cell samples were lysed in RIPA, and a BCA protein assay kit was used to determine the protein concentration. Briefly, the total protein lysates were separated on 12% SDS-PAGE gels for detecting Nrf2, Beclin1, LC3, and p62. Then the proteins were transferred to polyvinylidene difluoride (PVDF) membranes, blocked with 5% skimmed milk for 2 h at room temperature, and then immunoblotted with rabbit polyclonal anti-human antibodies against *β*-actin (1 : 1000), Nrf2 (1 : 1000), Beclin1 (1 : 1000), LC3 (1 : 1000), and p62 (1 : 1500) overnight at 4°C. This was followed by application of a goat anti-rabbit peroxidase conjugated secondary antibody (1 : 5000, Santa Cruz) for 2 h at 37°C. Then the bands were detected using the enhanced chemiluminescence system and Quantity One image analysis software was used to measure the band intensity. The housekeeping protein *β*-actin was used for loading control. For result analysis, we normalized the band intensity of target protein to *β*-actin in each sample, and then we normalized the relative target protein expression level of treated group to its control group.

### 2.7. Immunofluorescence

The cells were washed with phosphate-buffered saline (PBS) for three times, fixed with 4% paraformaldehyde for 20 min at room temperature, permeabilized with 0.5% Triton X-100 for 10 min, and then blocked with 1% bovine serum albumin (BSA) for 1 h at room temperature. Next, cells were incubated with rabbit polyclonal anti-human antibodies against Nrf2 (1 : 150), Beclin1 (1 : 150), and LC3 (1 : 150) at 4°C overnight, respectively. After that, cells were washed and incubated with goat anti-rabbit dylight 594 (red) IgG antibody (QENSHARE BIOLOGICAL Inc., Xi'an, China) or goat anti-rabbit FITC (green) IgG antibody (ZSGB-BIO Inc., Beijing, China) at 1 : 150 dilution for 60 min at room temperature. Nuclei were stained with 4′,6-diamidino-2-phenylindole for 5 min. Cells were visualized with the fluorescent microscope (Nikon Eclipse Ti-s, Japan) using appropriate excitation wavelength.

### 2.8. Autophagy Detection Using mRFP-GFP Adenoviral Vector

Ad-mRFP-GFP-LC3 was purchased from HanBio Technology Co. Ltd. (HanBio, shanghai, China) and the process of adenoviral infection was implemented according to the manufacturer's instructions. PANC-1 cells were plated in six orifice plates and determined to reach at the desired confluency of 50%–70% at the time of infection. Then, cells were cultured in DMEM supplemented with 2% FBS with the adenoviruses at a final MOI of 60 for 2 h at 37°C. After infection, cells were grown in medium with 10% FBS and used for further study at the time point of 48 h. Autophagy was observed under a fluorescence microscope (Nikon Eclipse Ti-s, Japan). Autophagic flux was determined by evaluating the number of GFP and RFP puncta (puncta/cell were counted).

### 2.9. Live Cell Microscopy and Imaging

For live cell imaging, PANC-1 cells infected with Ad-mRFP-GFP-LC3 were grown on glass-bottom dishes (MatTek). Then, the cells were transfected with Nrf2 siRNA. After transfection, cells were treated with ROS stimulation and observed by Live Cell Imaging Confocal Scanner System according to the manufacturer's instructions. Briefly, cells were maintained in a 5% CO_2_ chamber at 37°C through the acquisition. Three random positions of the cells were selected to monitor the process of autophagy. Cells were visualized with the fluorescent microscope using appropriate excitation wavelength, and images for the three selected positions were acquired at the beginning and the time point of 24 h.

### 2.10. Statistical Analysis

All experiments were repeated at least three times. Data are presented as Mean ± SD. Statistical analyses were performed using the Statistical Package for Social Science (SPSS) version 17.0 (SPSS Inc., Chicago, IL, USA). Student's *t*-test and one-way ANOVA with the LSD post hoc test were used to evaluate the differences of presented data. The overall *P* values presented here were two-sided. The significance level was set at a *P* value less than 0.05. In all figures, (**∗**) denotes *P* < 0.05.

## 3. Results

### 3.1. Expression of Nrf2 in Pancreatic Cancer Cell Lines

**Figure 1:**
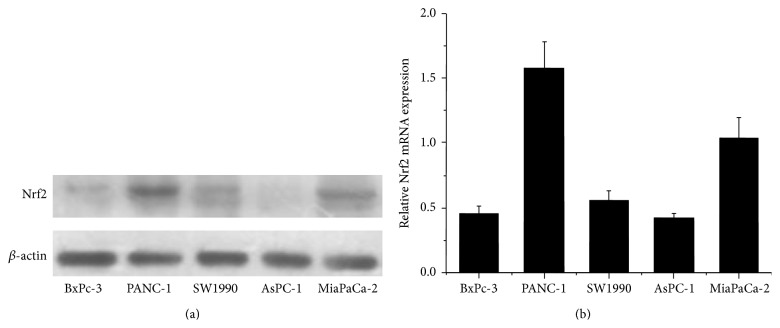
The expression of Nrf2 in pancreatic cancer cells. (a) The expression of Nrf2 at protein level in BxPc-3, PANC-1, SW1990, AsPC-1, and MiaPaCa-2 cells was evaluated by Western blotting. (b) The expression of Nrf2 mRNA level was estimated in 5 pancreatic cancer cell lines by qRT-PCR. The data are presented as Mean ± SD for three independent experiments. The bar graph below shows the relative mRNA expression levels among the cell lines. Column: Mean; bar: SD.

To explore the possible roles of Nrf2 pathway and autophagy under oxidative stress in pancreatic cancer cell lines, we first detected the expression of Nrf2 in 5 pancreatic cancer cell lines (BxPc-3, PANC-1, SW1990, AsPC-1, and MiaPaCa-2) by Western blot and real-time PCR analyses, respectively (Figures 1(a)-1(b)). We found that Nrf2 expression and transcription were the strongest in PANC-1 cells but the weakest in BxPc-3 and AsPC-1 cells.

### 3.2. Expression of Autophagic Related Protein Level in Pancreatic Cancer Cells

**Figure 2:**
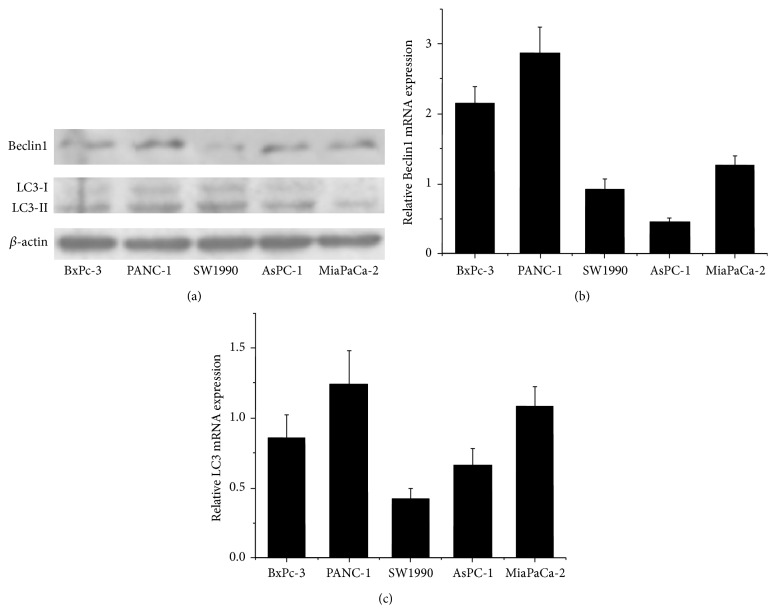
The expression of Beclin1 and LC3 in pancreatic cancer cells. (a) The expression of Beclin1 and LC3-II protein in BxPc-3, PANC-1, SW1990, AsPC-1, and MiaPaCa-2 cells was evaluated by Western blotting. (b-c) The expression of Beclin1 and LC3 mRNA level was estimated in 5 pancreatic cancer cell lines by qRT-PCR. The data are presented as Mean ± SD for three independent experiments. The bar graph shows the relative mRNA expression levels among the cell lines. Column: Mean; bar: SD.

Beclin1 and LC3 were conducted to detect the autophagic activity of pancreatic cancer cells for their important roles in autophagy. We detected the expression of Beclin1 and LC3 in 5 pancreatic cancer cell lines (BxPc-3, PANC-1, SW1990, AsPC-1, and MiaPaCa-2) by Western blot and real-time PCR analyses, respectively (Figures 2(a)–2(c)). We found that the expression of Beclin1 and LC3-II protein and also Beclin1 and LC3 transcription were all the strongest in PANC-1 cells. Therefore, we chose the cell line of PANC-1 for further study.

### 3.3. H_2_O_2_ Induces Increased Generation of ROS in PANC-1 Cells

To elevate the level of intracellular ROS in PANC-1 cells, exogenous hydrogen peroxide (H_2_O_2_) was used to treat cells. H_2_O_2_ is a prototypic reactive oxygen species (ROS) generated as a by-product of the normal oxidative metabolism. At low concentrations, H_2_O_2_ acts as a survival molecule, but at high concentrations it can lead to irreversible damage, followed by cell death. With the increased concentration of H_2_O_2_ in serum-free medium, the capacity of inhibiting proliferation for PANC-1 cells became more apparent. Ultimately, we selected 100 *μ*mol/L as the optimum concentration through the MTT assay (Figure  S1). Then, we observed that a large number of cancer cells died after 48 h when treated with 100 *μ*mol/L H_2_O_2_. Thus, we choose 24 h as the optimal H_2_O_2_ treated time. Flow cytometry was used to detect the intracellular ROS level in PANC-1 cells (Figure  S2). The results showed that a small amount of exogenous H_2_O_2_ can significantly improve the level of intracellular ROS.

### 3.4. The Influence of ROS on Nrf2 Expression and Autophagy in PANC-1 Cells

**Figure 3:**
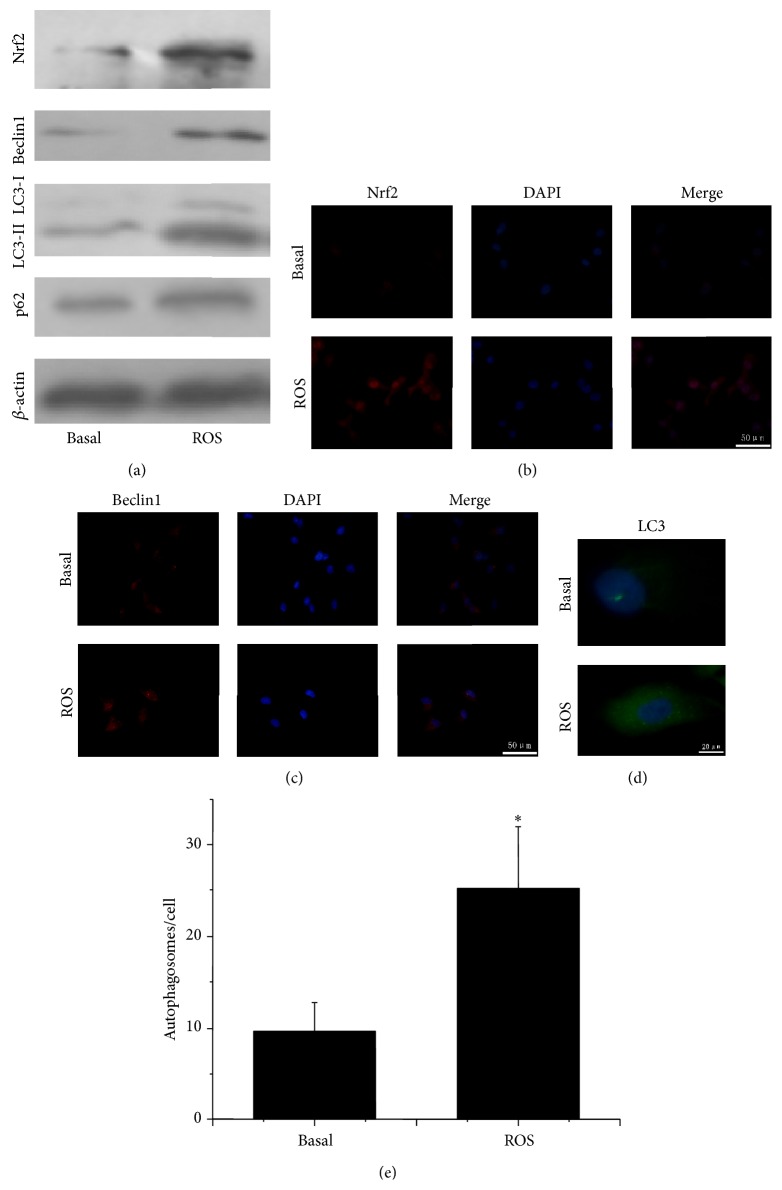
ROS stimulation induces translocation of Nrf2 to the nucleus and autophagy in PANC-1 cells. (a) The expression of Nrf2, Beclin1 and LC3-II and also the level of p62 in PANC-1 cells under basal condition and upon ROS stimulation. (b–d) Immunofluorescence images of PANC-1 cells for Nrf2, Beclin1 and LC3 under basal condition and upon ROS stimulation. (e) Numbers of autophagosomes per cell were counted in 10 random fields. The data are presented as Mean ± SD for three independent experiments. Column: Mean; bar: SD.

The expression of Nrf2 and autophagic related proteins were explored by Western blot. As shown in Figure 3(a), treatment with H_2_O_2_ (ROS) could significantly improve the expression of Nrf2, Beclin1, and LC3-II and also the level of p62 in PANC-1 cells. Immunofluorescence analyses indicated a marked increase of Nrf2 immunofluorescence signal in both the cytoplasm and the nucleus, suggesting that the expression of Nrf2 and nuclear translocation of Nrf2 were enhanced as an effect of increased ROS (Figure 3(b)). Furthermore, we found a stronger immunofluorescence signal for Beclin1 in the cytoplasm of cells upon ROS stimulation compared with control cells, indicating that the expression of Beclin1 was elevated by ROS (Figure 3(c)). Additionally, LC3 changed from the diffuse state to gathered granular in the cytoplasm (Figure 3(d)). We quantitated the occurrence of autophagy and found that autophagosomes were significantly promoted by ROS (Figure 3(e)). In addition, for the accumulation of p62 reflects the inhibition of autophagy, the increased level of p62 in our study indicated that ROS induces the p62 transcription.

### 3.5. The Influence of ROS on Nrf2 Expression in PANC-1 Cells Under Suppressed Autophagy

**Figure 4:**
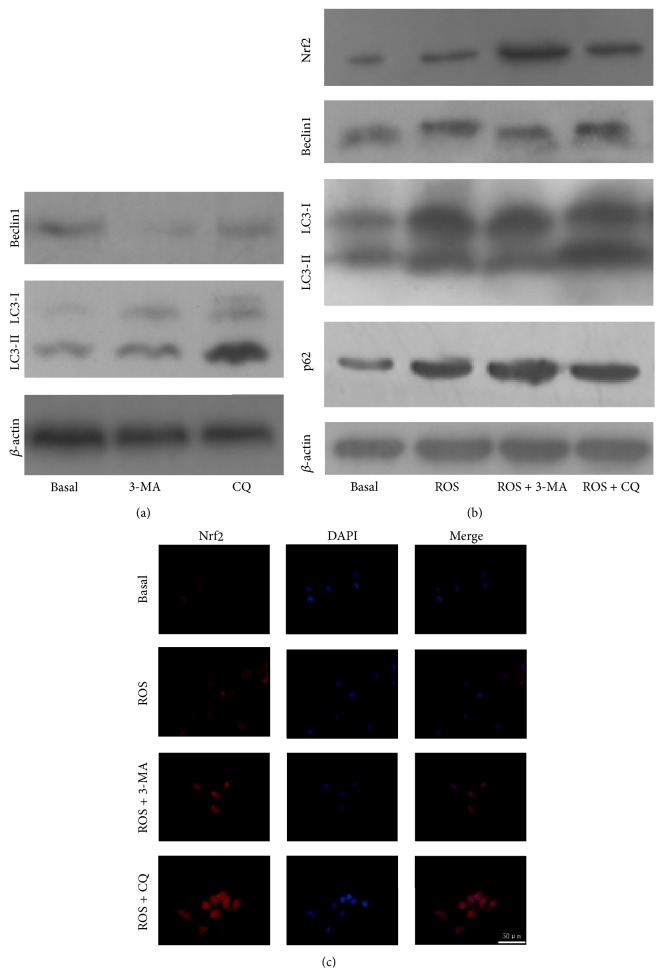
Suppression of autophagic activity enhances Nrf2 expression and translocation of Nrf2 to the nucleus upon ROS stimulation in PANC-1 cells. (a) The expression of Beclin1 and LC3-II treated with or without 3-MA or CQ in the absence of ROS. (b) The expression of Nrf2, Beclin1, and LC3-II and also the level of p62 in pancreatic cancer cells under basal condition and upon ROS stimulation with or without 3-MA or CQ. (c) Immunofluorescence images of PANC-1 cells for Nrf2 under basal condition and upon ROS stimulation with or without 3-MA or CQ.

To explore the effect of ROS on Nrf2 pathway when the autophagic activity was suppressed, we used different inhibitors (3-methyladenine: 3-MA and chloroquine: CQ) to prevent autophagy in PANC-1 cells at particular stages. 3-MA has been used to prevent the formation of autophagosomal precursors at the early stage of autophagy as an inhibitor of class III phosphatidylinositol 3-kinase and CQ, a lysosomotropic weak base, could inhibit the fusion of autophagosome with lysosome to prevent the process of autophagy [32]. As shown in Figure 4(a), we found that the treatment of 3-MA inhibited the expression of Beclin1, and CQ lead to accumulation of LC3-II in basal state. These results indicated that 3-MA and CQ both can inhibit the autophagic activity. Moreover, Western blot was used to detect the expression of Nrf2, Beclin1, and LC3-II and also the level of p62 when cells were under basal condition and upon ROS stimulation with or without 3-MA or CQ. We found that the treatment of 3-MA effectively arrested the ROS-induced autophagic activation determined by low Beclin1 expression and accumulation of p62. Similarly, CQ prevented the ROS-induced autophagy characterized as accumulation of LC3-II and p62 (Figure 4(b)).

Both inhibition and blockage of autophagy at particular stages caused a marked increase in the expression level of Nrf2 upon ROS stimulation (Figure 4(b)). Additionally, the expression of Nrf2 in the cytoplasm and the nuclear translocation of Nrf2 were both enhanced as an effect of autophagic inhibitor treatment upon ROS exposure, as demonstrated by immunofluorescence (Figure 4(c)). These findings indicated that suppression of autophagic activity results in an increased promotion of Nrf2 pathway upon ROS stimulation in PANC-1 cells.

### 3.6. The Influence of ROS on Autophagy in PANC-1 Cells Transfected with Nrf2 siRNA

**Figure 5:**
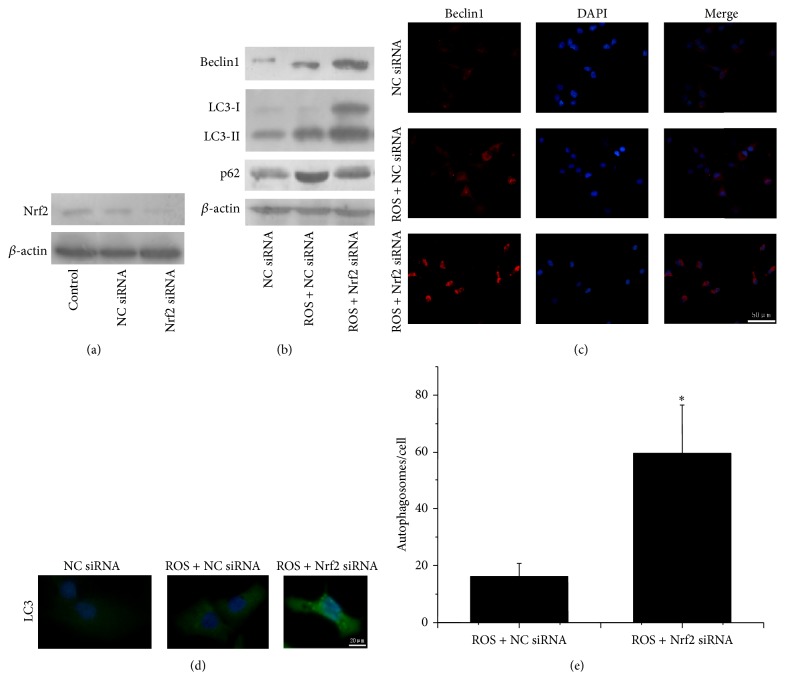
Silencing of Nrf2 promotes autophagy upon ROS stimulation in PANC-1 cells. (a) Western blot detection of Nrf2 in siRNA-transfected PANC-1 cells. (b) The expression of Beclin1 and LC3-II and also the level of p62 in Nrf2 siRNA-transfected PANC-1 cells upon ROS stimulation. (c-d) Immunofluorescence images of PANC-1 cells for Beclin1 and LC3 upon ROS stimulation. (e) Numbers of autophagosomes were counted in 10 random fields. The data are presented as Mean ± SD for three independent experiments. Column: Mean; bar: SD.

To confirm if Nrf2 pathway could regulate autophagy under the treatment of ROS, Nrf2 siRNA was applied to knock down Nrf2 in PANC-1 cells (Figure 5(a)). Using fluorescence microscope, we found that the transfection efficiency is up to 98% (Figure  S3A). Next, we screened appropriate siRNA sequence with RT-PCR and Western blot from six designed Nrf2 siRNAs. The result showed that the inhibition efficiency of NRF2-homo-1498 was the most efficient (Figure  S3B-C).

We detected the expression of autophagic related proteins when Nrf2 siRNA-PANC-1 cells were faced with ROS stimulation by Western blot and immunofluorescence. As shown in Figure 5(b), silencing of Nrf2 resulted in significant increase in the expression of Beclin1 and LC3-II after ROS intervention; similar results were detected using immunofluorescence (Figure 5(c)). Furthermore, we found a significant increase in gathered granular of LC3 in the cytoplasm when PANC-1 cells transfected with Nrf2 siRNA compared with NC siRNA upon ROS exposure, as demonstrated by immunofluorescence (Figure 5(d)). Additionally, we quantitated the occurrence of autophagy and found that autophagosomes were significantly promoted as a result of Nrf2 knockdown (Figure 5(e)).

P62, a link between LC3 and ubiquitinated substrates, could be successfully degraded by autophagy [33]. To determine whether silencing of Nrf2 promotes the exact autophagic flux of PANC-1 cells upon ROS stimulation, Western blot detection of p62 was used to assess the capacity of autophagic flux. As shown in Figure 5(b), the level of p62 protein was decreased as an effect of silencing of Nrf2 in PANC-1 cells upon ROS exposure. These findings indicated that silencing of Nrf2 promotes autophagic flux upon ROS stimulation in PANC-1 cells.

### 3.7. Silencing of Nrf2 in PANC-1 Cells upon ROS Stimulation Enhances Autophagic Flux by Promoting Autolysosome Formation

**Figure 6:**
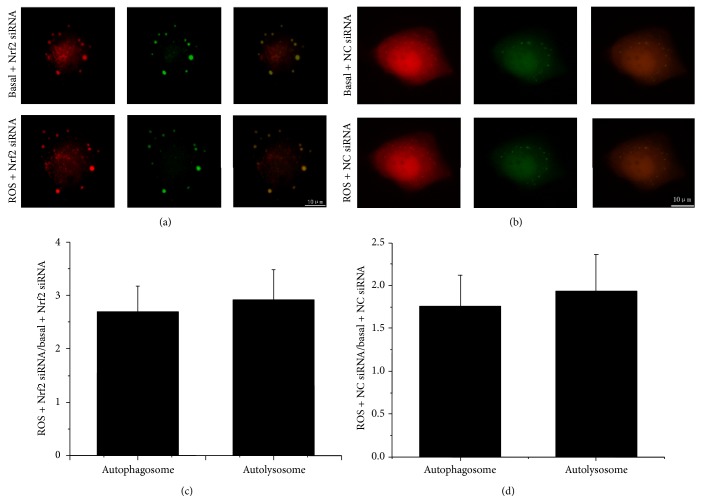
Silencing of Nrf2 in PANC-1 cells upon ROS stimulation promotes autolysosome formation. (a) PANC-1 cells were infected with the Ad-mRFP-GFP-LC3. Then, the cells were transfected with Nrf2 siRNA, treated with ROS stimulation, and observed by Live Cell Imaging Confocal Scanner System. (b) PANC-1 cells were infected with the Ad-mRFP-GFP-LC3. Then, the cells were transfected with NC siRNA, treated with ROS stimulation, and observed by Live Cell Imaging Confocal Scanner System. (c-d) Numbers of puncta in the same cell was determined when cells were under basal condition and upon ROS stimulation. Cells were counted in 10 random fields. The data of fold increase are presented as Mean ± SD for three independent experiments. Column: Mean; bar: SD.

As autophagy is a dynamic process, the detection of LC3 processing by Western blot and formation of autophagosomes by fluorescence to monitor autophagic activity is insufficient to determine the entire autophagic system. For example, the increased autophagosomes could suggest either autophagic activation or an inhibition of lysosomal degradation [34]. Therefore, to further confirm that the silencing of Nrf2 promotes the progression of complete process of autophagy (autophagic flux) upon ROS stimulation in PANC-1 cells, we observed live cells infected with the Ad-mRFP-GFP-LC3 to differentiate the autophagosome and autolysosome during autophagy. The assay takes advantage of the stability of red fluorescent protein (RFP) under acidic conditions and the acid-sensitive green fluorescent protein (GFP) and also the pH difference between autophagosome and autolysosome. Quenching of GFP and maintaining of RFP which exhibits red puncta could represent the autolysosome by indicating the fusion of autophagosome with acidic lysosomal compartment. However, maintaining of both GFP and RFP which exhibits yellow puncta could represent the autophagosome. Live Cell Imaging Confocal Scanner System was used to observe the autophagic flux of the same cells treated with or without ROS. As shown in Figures 6(a)-6(b), we found a successful introduction of this adenovirus displaying both fluorescent proteins when cells were infected with the Ad-mRFP-GFP-LC3. The results indicated marked fold increases of the autophagosomes and autophagosomes in both Nrf2 siRNA and NC siRNA-transfected PANC-1 cells when faced with ROS stimulation, but the Nrf2 siRNA-transfected PANC-1 cells tend to have the higher fold increase which suggests that inhibition of Nrf2 further promote the autophagic flux upon ROS stimulation (Figures 6(c)-6(d)). These results further confirmed that silencing of Nrf2 promotes autophagic flux upon ROS stimulation in PANC-1 cells.

## 4. Discussion

In the present study, we have provided direct evidence that Nrf2 pathway and autophagy have a negative interaction with each other in pancreatic cancer cells upon ROS stimulation. We have successfully set a model with ROS stress to better study the relevance of Nrf2 pathway and autophagy in pancreatic cancer cells.

It has been shown that high expression levels of Nrf2 have been observed in many cancers, including head and neck, gall bladder, lung, pancreas, and colorectal cancer [12, 14, 35, 36]. Moreover, the research of Yang et al. indicates a higher autophagic level in pancreatic cancer [6]. Nrf2 and autophagy both benefit the progression of pancreatic cancer. As we know, loss of autophagic function leads to accumulation of p62 which acts to sequester Keap1 into the autophagosomes, inhibiting the ubiquitylation of Nrf2, resulting in the noncanonical activation of Nrf2. However, it has been demonstrated that persistent activation of Nrf2 is critical for liver tumorigenesis that occur in mice with autophagy-deficient hepatocyte [37]. Moreover, Riley et al. consider that the accumulation of poly-Ub chains in circumstances with defects in autophagy is an indirect consequence of activation of Nrf2 [38]. Persistent activation of Nrf2 results in tumorigenesis, in this way, the Nrf2 inhibitors can be used to inhibit the persistent Nrf2 activation induced by loss of autophagic function to prevent the progression of pancreatic cancer. Meanwhile, a study showed that inhibition of autophagy leads to robust tumor regression and prolonged survival in pancreatic cancer xenografts and genetic mouse models. It seems that the inhibition of autophagy combined with Nrf2 inhibitors would be more effective to prevent the progression of pancreatic cancer due to its dark side of activating Nrf2 pathway.

Since the nutrient-limited environment in pancreatic cancer cells could lead to elevated ROS level, followed by activation of both Nrf2 pathway and autophagy, they may be involved in the protection of cells against oxidative stress. Low level of ROS could promote the antioxidant production and the tumor growth in cancer cells, but further increased production of ROS and/or decrease in antioxidant capacity would lead to the imbalance in oxidant-antioxidant system of cancer cells, resulting in cell death [39]. Some evidence indicated that Nrf2 has the ability to lower intracellular ROS by its antioxidant program [31]. For autophagy, its function of removing damaged organelles and accumulated proteins could prevent the further increase of ROS. Taken together, Nrf2 pathway and autophagy are required coordinately reducing the intracellular ROS accumulation to ensure the survival of pancreatic cancer cells, but their inner relationship is unclear. It is puzzling whether Nrf2 pathway or autophagy is the upstream regulator to modulate the other one. To make it clear, we first used exogenous H_2_O_2_ to improve the level of intracellular ROS, which resulted in activation of both Nrf2 pathway and autophagy. Then, for the several steps of autophagic process, different inhibitors (3-MA and CQ) were used to prevent autophagy at early or late stage, and the result showed that suppression of autophagic activity at different stages lead to an increased promotion of Nrf2 pathway. This indicates that when faced with oxidative stress, if the autophagic function of cancer cell is suppressed, the further activation of Nrf2 pathway is required to respond to excessive ROS to help cancer cell to survive.

On the other hand, we showed that silencing of Nrf2 resulted in significant increase in the expression of Beclin1 and LC3-II, which suggest that Nrf2 pathway regulates autophagy at both the initial and the final steps. As autophagy is a dynamic process, Western blot detection of p62 and Ad-mRFP-GFP-LC3 infection were used to monitor the change of autophagic flux after Nrf2 knockdown. Interestingly, our findings showed that silencing of Nrf2 promotes the exact autophagic flux. This indicates that when faced with oxidative stress, if the activation of Nrf2 pathway in cancer cell is repressed, the further promotion of effective autophagic flux is required to deal with the ROS mediated damage to avoid cell death. Thus, the relationship between Nrf2 pathway and autophagy cannot be a simple upstream or downstream. Taken together, when we regard ROS as the key factor, Nrf2 pathway and autophagy would be in a negative interaction with each other, and autophagy inhibition not only leads to accumulation of p62 to activate Nrf2 pathway by a noncanonical way but also increase ROS production to directly activate Nrf2 pathway.

Antioxidant programs inhibit excessive ROS production to maintain pancreatic cancer cells at quiescent state, causing chemotherapeutic and radio therapeutic resistance [40, 41]. We suggest that cancer cells with higher activated Nrf2 pathway and autophagy may have stronger ability to survive under oxidative stress due to their capacity to lower intracellular ROS. Combined with their negative interaction with each other, we suggest that novel drug targets for coinhibition of Nrf2 pathway and autophagy may be a potential therapy for preventing the progression of pancreatic cancer.

## 5. Conclusions

In summary, our findings suggest that Nrf2 pathway and autophagy have a negative interaction with each other upon ROS stimulation, and autophagy inhibition not only leads to accumulation of p62 to activate Nrf2 pathway by a noncanonical way but also increase ROS production to directly activate Nrf2 pathway. The demonstrated relationship between Nrf2 pathway and autophagy will advance our understanding of the progression of pancreatic cancer induced by ROS. Thus, novel drug targets for coinhibition of Nrf2 pathway and autophagy may be a potential therapy for preventing the progression of pancreatic cancer.

## Supplementary Material

Supplementary Table S1 presents the primers of Nrf2, Beclin1, LC3 and *β*-actin for Real-Time PCR.
Supplementary Table S2 provides six designed siRNAs for NRF2.
Supplementary Figure S1 shows the optimum H_2_O_2_ treated concentration for PANC-1 cells.
Supplementary Figure S2 presents the level of intracellular ROS of PANC-1 cells when treated with H_2_O_2_.
Supplementary Figure S3 shows the inhibition effects of designed Nrf2 siRNAs in PANC-1 cells.
