# Supplementary material for: The Relevance of Nrf2 Pathway and Autophagy in Pancreatic Cancer Cells upon Stimulation of Reactive Oxygen Species
Source: Oxid Med Cell Longev. 2015 Nov 22;2016:3897250. doi: 10.1155/2016/3897250 (PMC4670682; doi:10.1155/2016/3897250)
Supplement: Supplementary file 1 — Supplementary Table S1 presents the primers of Nrf2, Beclin1, LC3 and β-actin for Real-Time PCR. Supplementary Table S2 provides six designed siRNAs for NRF2. Supplementary Figure S1 shows the optimum H2O2 treated concentration for PANC-1 cells. Supplementary Figure S2 presents the level of intracellular ROS of PANC-1 cells when treated with H2O2. Supplementary Figure S3 shows the inhibition effects of designed Nrf2 siRNAs in PANC-1 cells. [file 3897250.f1.docx]

**Table S1:** Primers for Real-Time PCR

| **genes** | **forword primer** | **reverse primer** |
| --- | --- | --- |
| Nrf2 | 5′-CCAACACACGGTCCACAGCT-3′ | 5′-TCCGTCGCTGACTGAAGTCAA-3′ |
| Beclin1 | 5′-GAACCGCAAGATAGTGGC-3′ | 5′-CAGAGCATGGAGCAGCAA-3′ |
| LC3 | 5′-GAGCAGCATCCAACCAAA-3′ | 5′-CGTCTCCTGGAGGCATA-3′ |
| β-actin | 5′-AGCTACGAGCTGCCTGACG-3′ | 5′-GCATTTGCGGTGGACGAT-3′ |

**Table S2:** siRNA for NRF2

| **siRNA** | **sequence** |
| --- | --- |
| NRF2-homo-624 | 5’-GGAGGCAAGAUAUAGAUCUTT-3’  5’-AGAUCUAUAUCUUGCCUCCTT-3’ |
| NRF2-homo-1624 | 5’-CCAGAACACUCAGUGGAAUTT-3’  5’-AUUCCACUGAGUGUUCUGGTT-3’ |
| NRF2-homo-2081 | 5’-GCUGCUCAGAAUUGCAGAATT-3’  5’-UUCUGCAAUUCUGAGCAGCTT-3’ |
| NRF2-homo-934 | 5’-CCCGUUUGUAGAUGACAAUTT-3’  5’-AUUGUCAUCUACAAACGGGTT-3’ |
| NRF2-homo-1498 | 5’-GCCCAUUGAUGUUUCUGAUTT-3’  5’-AUCAGAAACAUCAAUGGGCTT-3’ |
| NRF2-homo-2226 | 5’-GCACCUUAUAUCUCGAAGUTT-3’  5’-ACUUCGAGAUAUAAGGUGCTT-3’ |


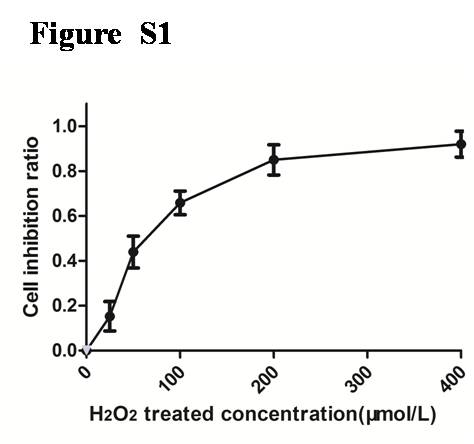


**Figure S1:** MTT assays were performed to determine the optimum H_2_O_2_ treated concentration for PANC-1 cells.

**Figure S2:** Exogenous H_2_O_2_ improves the level of intracellular ROS of PANC-1 cells. Intracellular ROS level was represented by the DCF intensity and was detected by flow cytometry. The data are presented as Mean ± SD for three independent experiments. (a) H_2_O_2_ treated concentration: 0μmol/L; (b) H_2_O_2_ treated concentration: 100μmol/L. Column: Mean; bar: SD.

**Figure S3:** The inhibition effects of designed Nrf2 siRNAs in PANC-1 cells. (A) The [transfection](javascript:void(0);) [efficiency](javascript:void(0);) of siRNA was monitored by fluorescence microscope. (B) The expression of Nrf2 mRNA level was estimated in PANC-1 cells by RT-PCR after transfected with designed Nrf2 siRNAs. (C) The expression of Nrf2 protein was evaluated in PANC-1 cells by Western blotting after transfected with designed Nrf2 siRNAs.
